# Association between eosinophilia and renal prognosis in patients with pathologically proven cholesterol crystal embolism

**DOI:** 10.1007/s10157-020-01886-9

**Published:** 2020-04-07

**Authors:** Yasuhiro Mochida, Takayasu Ohtake, Kunihiro Ishioka, Machiko Oka, Kyoko Maesato, Hidekazu Moriya, Sumi Hidaka, Shuzo Kobayashi

**Affiliations:** Kidney Disease and Transplant Center, Shonan Kamakura General Hospital, 1370-1 Okamoto, Kamakura, Kanagawa 247-8533 Japan

**Keywords:** Cholesterol crystal, Embolism, Eosinophilia, Renal prognosis, Dialysis

## Abstract

**Background:**

Approximately, 20–70% of patients with cholesterol crystal embolism (CCE) have eosinophilia. However, it remains unknown how eosinophilia influences renal prognosis in patients with CCE. In this study, we investigated the association between eosinophil count (Eo) and renal prognosis in CCE patients on steroid therapy.

**Methods:**

The present study is a single-centered retrospective cohort study in patients with renal dysfunction and CCE from April 2007 to May 2018. This study included the patients who were treated with neither maintenance dialysis nor steroid before CCE diagnosis, and followed-up for kidney function until November 2019. We assessed whether eosinophilia at the time of CCE diagnosis was related to renal death after treating with steroid therapy.

**Results:**

Thirty patients with pathologically diagnosed CCE were enrolled and followed-up for 11.0 (5.2–43.4) months. There were significant differences in the white blood cell count (*p* = 0.01), hemoglobin (*p* = 0.009), serum creatinine levels (*p* = 0.008), phosphate (*p* = 0.049), and Eo (*p* = 0.008) between the renal survival and renal death groups. Using the receiver operating characteristic curve analysis with Youden index, Eo of 810/µL showed 100% sensitivity and 69.6% specificity for detecting renal death (area under the curve: 0.839). Comparing the outcomes in patients having Eo ≥ and < 810/µL using the log-rank test, there is a significantly higher renal death rate in CCE patients with Eo ≥ 810/µL (*p* = 0.0016).

**Conclusion:**

Higher eosinophilia was a prognostic risk factor for renal death in the patients with CCE.

## Introduction

Flory et al*.* were the first to have studied cholesterol crystal emboli (CCE). They reported that the autopsy performed in men with advanced arteriosclerosis showed numerous occlusions of small arteries with cholesterol crystals in multiple organs, such as the kidney, spleen, pancreas, and thyroid [1]. CCE occurs following the disruption of an aortic intimal plaque with the crystals being systematically dispersed in multiple organs. The cause of CCE is largely iatrogenic and the condition is often associated with invasive procedures in vessels, such as aortic surgery, coronary and abdominal angioplasty. Patients with CCE may have several clinical characteristics including eosinophilia, blue toes, warfarin use, and renal dysfunction after invasive procedure.

Some studies have reported that about 20–70% of patients with CCE have eosinophilia (> 500/µL) [2–7]. However, the influence of eosinophilia on the prognosis has not been studied. The disease and syndrome associated with eosinophilia include eosinophilic granulomatosis with polyangiitis, chronic eosinophilic pneumonia, and hypereosinophilic syndrome, etc. In all these disorders, eosinophilic inflammation is a predominant pathogenic feature and leads to multiorgan damage. Herein, we hypothesize that CCE patients with eosinophilia have associated with more severe kidney damage. In this study, we investigated the association between eosinophilia and the renal prognosis in CCE patients.

## Materials and methods

### Participants

#### Study patients

This was a single-centered retrospective cohort study of patients who had the biopsy-proven CCE at the Shonan Kamakura General Hospital from April 2007 to May 2018. Patients were followed-up until November 2019 and information was collected with regard to laboratory tests, renal replacement therapy, and all-cause mortality from the electronic records. All patients were consulted to nephrologists for renal dysfunction, diagnosed CCE pathologically, and treated with steroid therapy in this study. We excluded those patients on maintenance renal replacement therapy prior to the diagnosis of CCE and the patients not being given steroid therapy following the diagnosis of CCE. The study was approved by the Ethical Committee of the Tokushukai Group Institutional Review Board (Approval number IRB No. TGE01161-024) and was conducted as per the guidelines of the declaration of Helsinki. For this study, we adopted an opt-out consent design instead of the written informed consent. We provided patients with information explaining the proposed research project (the purpose, required individual data, and duration of research) by means of an information sheet or the website of the hospital, and gave them the opportunity to opt-out.

#### Definition of CCE and renal death

Biopsy-proven typical pathological finding (the cholesterol creft) was the definition of CCE in this study. Renal death was defined as the requirement of maintenance hemodialysis.

#### Measurements

From the electronic medical records, we obtained information about baseline patient characteristics and the use of medications (antihypertensives, anti-platelet, and anti-coagulation drugs). Baseline characteristics included age, sex, smoking, comorbidity [hypertension (HT), dyslipidemia, diabetic mellitus (DM), ischemic heart disease (IHD), stroke, and peripheral artery disease (PAD)]. Body mass index (BMI) was calculated from the weight and height measurements as weight (kg) divided by the square of the height (m^2^). Blood samples were obtained before initiating steroid therapy. Cell blood count (CBC), eosinophil count (Eo), hemoglobin (Hb), albumin, blood urea nitrogen (BUN), serum creatinine, estimated glomerular filtration rate (eGFR), calcium, phosphate, C-reactive protein (CRP), total cholesterol (T-cho), triglyceride (TG), high density lipid (HDL), low density lipid (LDL), and hemoglobinA1c (HbA1c) were assessed. Urine samples were also obtained at the same time. We also investigated the presence of known factors contributing to the formation of CCE, such as past history of cardiovascular interventions, operations, and aneurysms in the aorta between the thoracic arch and the division into iliac arteries. The dose and duration of steroid therapy were noted from the records. The interval period between invasive procedure and diagnosis of CCE was also investigated.

#### Study end points

The primary end point was whether eosinophilia was related to renal death. We initiated steroid therapy following the diagnosis of CCE. Serum creatinine levels were followed-up at 1, 3, 6, and 12 months, and every 6 months thereafter, after the initiation of steroid therapy. The patients were followed-up to check whether they initiated maintenance renal replacement therapy.

#### Secondary end points

In addition, we analyzed whether any difference in renal death was seen among the four groups: with/without aortic aneurysms (thoracic or abdominal) and/or eosinophilia, and whether the number of eosinophils had any correlation with the interval between performance of invasive procedures and diagnosis.

### Statistical analysis

Continuous data are expressed as median [interquartile range (IQR)], and categorical data are expressed as numbers (%). To determine the factor of worsening renal dysfunction, we divided patients into two groups, i.e., renal survival and renal death group, and compared the Student t test or Fisher exact test among these groups. We analyzed the validity of eosinophil counts using receiver operating characteristic (ROC) curve analysis. The Youden index was used to determine cut-off values of the eosinophil counts for predicting renal death. Renal death was calculated with the Kaplan–Meier method, and comparisons between two different eosinophil groups were made by the log-rank test. Proportion of renal death was compared with groups between eosinophilia and/or aortic aneurysm by Fisher exact test. A value of *p* < 0.05 was considered to indicate significance. All statistical analyses were done with IBM SPSS Statistics Software Ver.21 (SPSS Inc. Chicago, Ill, USA).

## Results

### Patient characteristics (Table 1)

**Table 1:** Baseline patient characteristics

	All patients	Renal survival	Renal death	*p*
*n*	30	23	7	
Demographic				
Age (years)	77 [73, 80]	77 [73, 81]	75 [71, 79]	0.12
Male (%)	29 (96.7)	22 (95.7)	7 (100)	1
Smoking (%)	24 (80.0)	19 (82.6)	5 (71.4)	0.6
Comorbidity				
HT (%)	30 (100)	23 (100.0)	7 (100.0)	1
Dyslipidemia (%)	21 (70.0)	16 (69.6)	5 (71.4)	1
DM (%)	12 (40.0)	8 (34.8)	4 (57.1)	0.39
IHD (%)	20 (66.7)	15 (65.2)	5 (71.4)	1
PAD (%)	9 (30.0)	5 (21.7)	4 (57.1)	0.15
Stroke (%)	7 (23.3)	6 (29.2)	1 (25.0)	1
Abdominal and/or thoracic aortic aneurysm (%)	20 (66.7)	15 (65.2)	5 (71.4)	1
Invasive procedure				
Invasive procedures of vessels (%)	19 (63.3)	15 (60.9)	5 (71.4)	0.68
Idiopathic (%)	11 (36.7)	9 (37.5)	2 (25.0)	0.68
Period between procedure and diagnosis(day)	60 (45, 127)	55 (30. 90)	45 (30, 75)	0.5
Medications				
ACEi (%)	2 (6.7)	1 (4.2)	1 (12.5)	0.44
ARB (%)	20 (66.7)	15 (62.5)	7 (87.5)	0.38
ACEi or ARB (%)	22 (73.7)	16 (66.7)	8 (100)	0.08
β or αβ blocker (%)	13 (43.3)	11 (45.8)	3 (37.5)	1
Ca blocker (%)	18 (60.0)	15 (62.5)	4 (50.0)	0..68
Aldosterone blocker (%)	5 (16.7)	3 (12.5)	2 (25.0)	0.58
Diuretics (%)	12 (40.0)	7 (29.2)	6 (75.0)	*0.038
Statin (%)	20 (66.7)	17 (70.8)	5 (62.5)	0.68
Anticoagulation therapy (%)	4 (13.3)	3 (12.5)	1 (12.5)	1
Antiplatelate therapy (%)	22 (73.3)	17 ( 70.8)	7 ( 87.5)	0.642
Clinical findings				
Blue toe (%)	26 (86.7)	19 (79.2)	7 (87.5)	1
CCE with blue toe at skin biopsy (%)	23/24 (95.8)	18/19 (94.7)	6/6 (100)	0.58
Retina CCE (%)	6 (25.0)	5 (20.8)	3 (37.5)	0.63
Renal CCE, n / kidney biopsy, n (%)	9/11 (81.8)	6/8 (75)	3/3 (100)	0.79
Gastrointestinal tract	2 (6.7)	0	2 (28.6)	0.06
Laboratory data				
WBC (/µL)	8050 [5900, 9600]	7600 [5750, 8750]	11,800 [9350, 12350]	*0.01
Eosinophil count (/µL)	740 [487, 1041]	560 [433, 919]	1200 [938, 1750]	*0.007
Hb (g/dl)	9.9 [9.0, 10.9]	10.3 [9.8, 11.4]	8.7 [8.1, 9.4]	*0.009
Plt (× 10^4^ /µL)	18.9 [13.4, 20.4]	19.2 [14.3, 20.5]	16.6 [12.9, 19.6]	0.42
Alb (g/dL)	3.6 [3.2, 3.9]	3.6 [3.3, 3.9]	3.5 [3.3, 3.9]	0.9
BUN (mg/dL)	62.3 [37.2, 70.0]	49.2 [36.0, 68.8]	65.7 [56.9, 101.3]	0.07
Cr (mg/dL)	4.42 [3.23, 5.78]	3.75 [3.12, 5.22]	6.98 [5.39, 7.62]	*0.008
eGFR (ml/min/1.73m^2^)	11.4 [8.4, 15.3]	12.0 [9.4, 16.2]	6.7 [5.8, 8.5]	*0.009
Ca (mg/dL)	8.9 [8.6, 9.3]	8.7 [8.6, 9.4]	9.0 [8.8, 9.1]	0.84
P (mg/dL)	4.2 [3.2, 4.9]	3.6 [3.2, 4.7]	5.4 [4.5, 5.7]	*0.049
Ca × P	37.1 [29.0, 44.2]	34.9 [27.3, 41.1]	45.9 [39.67, 52.4]	0.053
CRP (mg/dL)	0.91 [0.31, 2.72]	0.69 [0.24, 2.85]	0.63 [1.18, 7.25]	0.07
HbA1c (%)	5.70 [5.60, 6.00]	5.9 [5.6, 6.1]	5.7 [5.6, 5.7]	0.21
TCHO (mg/dL)	1820 [156, 226]	180 [159, 214]	210 [153, 249]	0.7
TG (mg/dL)	129 [96, 167]	131 [93, 165]	113 [99, 165]	0.88
HDL (mg/dL)	44 [40, 55]	46 [43, 58]	41 [32, 44]	0.12
LDL (mg/dL)	99 [87, 128]	103 [85, 127]	92 [91, 122]	0.88
LDL/HDL	2.21 [1.79, 3.01]	2.16 [1.76, 2.61]	2.94 [1.99, 3.64]	0.36
U-TP (g/day)	0.32 [0.14, 0.95]	0.18 [0.07, 0.74]	0.35 [0.07, 0.47]	0.73
Treatment, period and results				
PSL/wight (mg/kg)	0.35 [0.30, 0.43]	0.33 [0.30, 0.40]	0.42 [0.34, 0.47]	0.14
Period on PSL (month)	6.0 [3.0, 19.3]	9.0 [3.5, 23.0]	5.0 [3.0, 6.5]	0.17
Observational period (month)	11.0 [5.2, 43.4]	15.3[4.3, 43.9]	7.1 [5.9, 10.3]	0.54
Death (%)	13 (43)	8 (34.8)	5 (71.4)	0.19

*Alb* albumin, *ACEi* angiotensin converting enzyme inhibitor, *ARB* angiotensin II receptor blocker, *BUN* blood urine nitrogen, *Ca* calcium, *CBC* cell blood count, *Cr* creatinine, *CRP* C-reactive protein, *DM* diabetic mellitus, *eGFR* estimated glomerular filtration rate, *Hb* hemoglobin, *HbA1c* hemoglobin A1c, *HDL* high density lipoprotein, HT hypertension, *IHD* ischemic heart disease, *LDL* low density lipoprotein, *P* phosphate, *PAD* peripheral artery disease, *PLT* platelet, *PSL* prednisolone, *TCHO* total cholesterol, *TG* triglyceride, *TP* total protein, *WBC* white blood cell

*p value < 0.05

Based on the inclusion and exclusion criteria, 30 patients were finely enrolled. Table 1 shows the baseline characteristics of the patients in our study. The median age was 77 [73, 80] years [IQR]. Hypertension, diabetes mellitus, and ischemic heart disease were seen in 30 (100%), 12 (40%), and 20 (66.7%) patients, respectively. Iatrogenic CCE following procedures such as angiography and/or cardiovascular surgery was seen in 19 patients (63.3%), and 12 of these 19 patients (63.2%) had an abdominal aortic aneurysm (AAA) and/or a thoracic aortic aneurysm (TAA). Of the 11 patients with idiopathic (negative invasive procedure) CCE, 8 patients (72.7%) had AAA and/or TAA (Table 1b). Among the 30 patients with CCE, 4 (13.3%) were on anticoagulant therapy and 20 (65.7%) patients were on statin therapy. Blue toes were seen in 26 patients (86.7%), and of these, 24 patients received a skin biopsy; cholesterol clefts in the dermis were confirmed in 23 of these 24 patients (96%). In the remaining 7 patients who did not diagnose by skin biopsy, all of them had renal cholesterol embolisms. The mean Eo, Hb, and serum creatinine were 740 [487, 1041]/µL, 9.9 [9.0, 10.9] g/dL, and 4.42 [3.23, 5.78] mg/dL, respectively. The dose of steroid was 0.3–0.5 mg/kg in all patients and the duration of steroid therapy was for 6.0 [3.0, 19.3] months. Observational period was 11.0 [5.2, 43.4] months. Between the renal survival and renal death groups, there were significant differences in white blood cell (*p* = 0.01), hemoglobin (*p* = 0.007), serum creatinine levels (*p* = 0.009), phosphate (*p* = 0.049), and Eo (*p* = 0.007).

### Changes in renal function (Fig. 1)

**Fig. 1:**
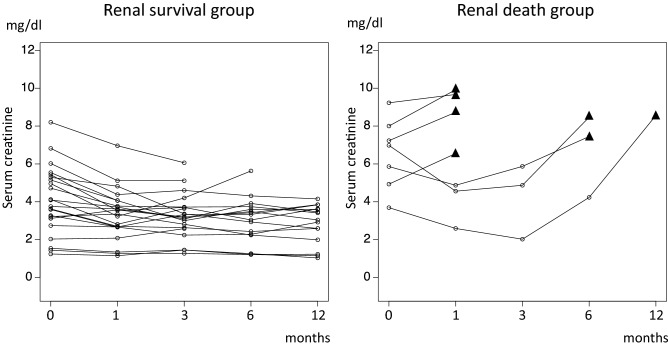
Changes in renal function in the renal survival group and renal death group for 12 months. Black triangle (▲) in renal death group showed the timing of starting dialysis

Figure 1 shows the trend of serum creatinine levels in the renal survival and renal death groups for 1 year after steroid therapy. Within 12 months, all patients in renal death group had maintenance renal replacement, and 3 of them showed improved transient renal function after starting steroid therapy, regardless of their poor renal function status.

### Renal death

#### ROC analysis and Kaplan–Meier curve (Fig. 2)

**Fig. 2:**
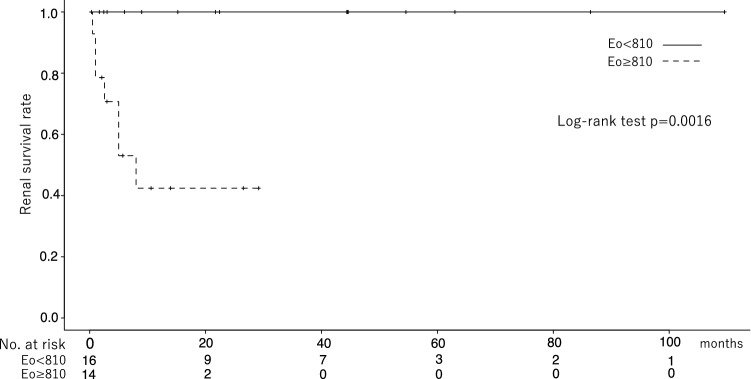
Kaplan–Meier curve. Comparisons between two different eosinophil groups (≥ and < 810/μL) were made by log-rank test. Eosinophilia ≥ 810/µL significant group associated with renal death (*p* = 0.0016)

We analyzed the validity of Eo for renal death using the ROC curve analysis with Youden index. As a result, when the cut-off value of Eo was set at 810/µL, the sensitivity and specificity for predicting renal death were 100% and 69.6%, respectively (area under the curve: 0.839). Comparing the outcomes in patients with Eo < 810/µL and ≥ 810/µL and group using the log-rank test, CCE patients with Eo ≥ 810/µL had a greater risk of renal death (*p* = 0.0016, Fig. 2).

#### Relation to renal death between aortic aneurysm and eosinophilia (Fig. 3)

**Fig. 3:**
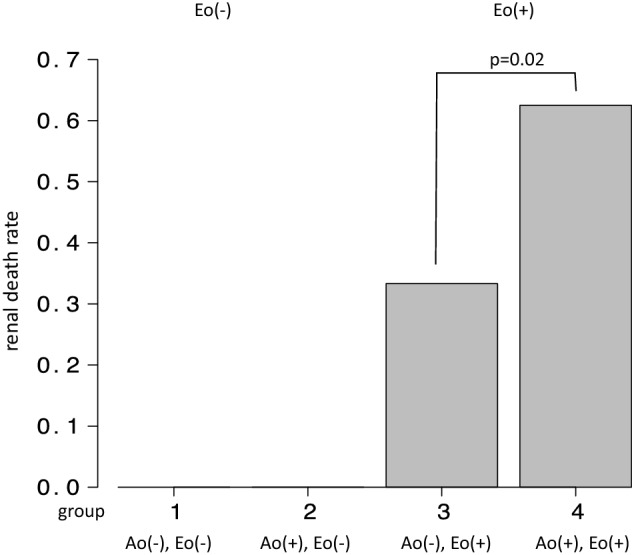
Renal death according to aneurysm and eosinophilia (≥ 810 µL). We analyzed the ratio of renal death between the following four groups: group 1: no aneurysm and eosinophil count (Eo) < 810/µL, group 2: positive for aneurysm and Eo < 810/µL, group 3: no aneurysm and Eo ≥ 810/µL, group 4: positive for aneurysm and Eo ≥ 810/µL. Group 4 showed significantly higher renal death rate compared to *p* < 0.05. A (−)—no aortic aneurysm, A (+)—positive aortic aneurysm, Eo (−)—eosinophil count < 810/µL, Eo( +)—eosinophil count ≥ 810µL

We analyzed whether there was any difference in renal death between the 4 groups, i.e., with/without aneurysm and/or eosinophilia (< 810/µL and 810/µL ≤), using the Fisher exact test. Group 4 (with aneurysm and Eo ≥ 810/µL) showed significant renal death than other groups (*p* < 0.05).

#### Correlation with eosinophil number and interval period from performance of invasive procedures to diagnosis

The interval period from the performance of invasive procedure to the diagnosis of CCE was not different between the renal survival and renal death groups (*p* = 0.50). The number of eosinophils did not correlate with the interval period between performance of the invasive procedures and the baseline (*p* = 0.73, *r* = − 0.08, *n* = 19).

## Discussion

We hypothesized that CCE patients with eosinophilia have more severe kidney damage and bring a poor prognosis. Therefore, we investigated an association between eosinophilia and renal prognosis. Our results showed that not only serum creatinine, but also eosinophilia was associated with renal death in patients with CCE.

Review of past literatures showed that end-stage renal disease (ESRD) in patients with atheroembolic renal disease is associated independently with long-standing hypertension [8], pre-existing chronic renal impairment [6, 8], diabetes mellitus [6], and heart failure [6]. Statins have been shown to delay the onset of ESRD [6, 8]. Eosinophilia has been defined as an eosinophil count of > 500/μL in many instances, and the risk factors for renal death have been investigated in situations where the patients were not on steroid therapy. The reported eosinophilia count might be biased in these situations. However, in our study, all the patients were prescribed steroid therapy, and the association between eosinophilia (> 810/µL) and renal prognosis was analyzed. As a result, we showed that eosinophilia was associated with the renal prognosis in patient with CCE.

Eosinophils are activated by the mediators, including interleukin IL-3, IL-5, and granulocyte–macrophage colony-stimulating factor (GM-CSF), followed by an increase in the circulation for 18–24 h before migrating to the extravascular tissue. Then, the activated eosinophil infiltrate and damage tissues by the following mechanisms: (1) release of granule products (major basic protein, eosinophil cationic protein, eosinophil-derived neurotoxin, and eosinophil peroxidase) damage the epithelial tissue and induce the inflammatory cells: macrophage and lymphocytes, (2) release of platelet activating factor, cytokines, and chemokines [GM-CSF, transforming growth factor beta (TGF-B), etc.] trigger tissue remodeling and fibrosis [9]. Eosinophils show the highest inflammatory activity when GM-CSF is released. Makiya et al. reported that the eosinophil count and the CD69 expression correlated with the granule protein concentration [10]. Liapis et al. showed that activated eosinophils contributed to thrombotic microangiopathy (TMA) [11]. Thus, the eosinophil count might influence the severity of inflammation.

Although Nakayama et al. showed the efficacy of low dose steroids [12] and steroids with statins [13], we evaluated why steroid therapy was not effective in patients with high eosinophilia, even though the counts decreased in all patients in our study. The eosinophil count might influence not only the severity, but also the volume of cholesterol embolism because the eosinophil count could be a reflection of the mechanism that induces the rejection of the cholesterol crystal, as well as the parasite elimination causes nucleotide-binding domain and leucine-rich repeat-containing pyrin domain containing receptor 3 (NLRP3) inflammasome activation, IL-1β, and eosinophilia [14]. In fact, cholesterol crystals are formed in the early atherosclerotic process and activate the inflammasome NLRP3 leading to the secretion of caspase-1 activation and IL-1β [15], which influence the release of mast cells and eosinophils [16–18]. Thus, we hypothesize that an increase in the number of scraping cholesterol crystals may result in eosinophilia and non-reversible renal failure.

To avoid the worsening of renal failure, we must pay careful attention to the eosinophil count in patients with arteriosclerosis, and especially chronic kidney disease (CKD) with aortic aneurysm and/or following endovascular procedures. CKD patients are seen to have a high risk of atherosclerosis and cardiovascular events [19, 20], and the risk of renal death increased in these patients when they also had CCE (5, 7). Ishi et al. investigated the association between CKD stage and eosinophil count among cardiac patients. They concluded that the eosinophil count was positively associated with more advanced CKD stages among cardiac patients, some of which might be related to subclinical cholesterol embolization [21]. Recently ruptured aortic plaques, which might cause atheromatous embolization, are thought to be not mainly iatrogenic, but spontaneous and continuously ongoing. Komatsu et al. showed that spontaneous ruptured aortic plaques (SRAP) are present in about 86.4% of patients who were suspected to have stable angina with no aneurysms, using nonobstructive angioscopy (NOA) [22]. Therefore, in patients with aneurysms with increased number of thrombi, NOA might help to identify a greater number of SRAP. In our study, the CCE patients with eosinophilia and aneurysm had a worse renal prognosis compared with the other groups. Thus, we consistently need to investigate eosinophil count in patients not only after endovascular operations, but also in those with CKD and/or aneurysms.

Our study has some limitations. First, our study was a retrospective study, and the protocol of steroid therapy for each patient varied with regard to the dose or duration of corticosteroids. Therefore, the effect of steroid therapy on renal outcome could be biased. However, the dose and duration of steroid therapy were not significantly different between the renal survival and death groups. Second, we did not measure the markers that influenced eosinophil activity, such as IL-3, IL-5, and GM-CSF, and were unable to identify the association between eosinophilia and the extent of eosinophilic inflammation. Finally, our study comprised a small population group, and multivariate analysis could not be done for small number of patients. Thus, larger prospective studies, including a randomized controlled trial, will be needed to evaluate the efficacy of early steroid therapy on the renal outcome in CCE patients before the eosinophil count raises over 810/µL.

## Conclusion

In conclusion, higher eosinophil counts might trigger renal death in patients with CCE. Therefore, the eosinophil count in patients with arteriosclerosis needs to be carefully monitored, especially in CKD with aortic aneurysm and/or following endovascular operations.

## Abbreviation

AAA: Abdominal aortic aneurysm
Alb: Albumin
ACEi: Angiotensin converting enzyme inhibitor
ARB: Angiotensin II receptor blocker
BUN: Blood urine nitrogen
Ca: Calcium
CBC: Cell blood count
CKD: Chronic kidney disease
CCE: Cholesterol crystal embolism
Cr: Creatinine
CRP: C-reactive protein
DM: Diabetic mellitus
eGFR: Estimated glomerular filtration rate
ESRD: End-stage renal disease
GM-CSF: Granulocyte–macrophage colony-stimulating factor
Hb: Hemoglobin
HbA1c: Hemoglobin A1c
HDL: High density lipoprotein
HT: Hypertension
HR: Hazard ratio
IHD: Ischemic heart disease
IL: Interleukin
IQR: Interquartile range
LDL: Low density lipoprotein
NLRP3: Nucleotide-binding domain and leucine-rich repeat-containing pyrin domain containing receptor 3
P: Phosphate
PAD: Peripheral artery disease
PLT: Platelet
PSL: Prednisolone
ROC: Receiver operating characteristic
RRT: Renal replacement therapy
TAA: Thoracic aortic aneurysm
TCHO: Total cholesterol
TG: Triglyceride
TGF: Transforming growth factor beta
TP: Total protein
TMA: Thrombotic microangiopathy
WBC: White blood cell
